# HCV-related burden of disease in Europe: a systematic assessment of incidence, prevalence, morbidity, and mortality

**DOI:** 10.1186/1471-2458-9-34

**Published:** 2009-01-22

**Authors:** Nikolai Mühlberger, Ruth Schwarzer, Beate Lettmeier, Gaby Sroczynski, Stefan Zeuzem, Uwe Siebert

**Affiliations:** 1 Institute of Public Health, Medical Decision Making and Health Technology Assessment, Department of Public Health, Information Systems and Health Technology Assessment, UMIT - University of Health Sciences, Medical Informatics and Technology, Hall i.T, Austria; 2 Department of Internal Medicine, Gastroenterology, Hepatology, Pneumology and Endocrinology, Johann Wolfgang Goethe-University, Frankfurt a.M, Germany; 3 Institute for Technology Assessment and Department of Radiology, Massachusetts General Hospital, Harvard Medical School, Boston, MA, USA; 4 Program in Health Decision Science, Department of Health Policy and Management, Harvard School of Public Health, Boston, MA, USA

## Abstract

**Background:**

Hepatitis C virus (HCV) is a leading cause of chronic liver disease, end-stage cirrhosis, and liver cancer, but little is known about the burden of disease caused by the virus. We summarised burden of disease data presently available for Europe, compared the data to current expert estimates, and identified areas in which better data are needed.

**Methods:**

Literature and international health databases were systematically searched for HCV-specific burden of disease data, including incidence, prevalence, mortality, disability-adjusted life-years (DALYs), and liver transplantation. Data were collected for the WHO European region with emphasis on 22 countries. If HCV-specific data were unavailable, these were calculated via HCV-attributable fractions.

**Results:**

HCV-specific burden of disease data for Europe are scarce. Incidence data provided by national surveillance are not fully comparable and need to be standardised. HCV prevalence data are often inconclusive. According to available data, an estimated 7.3–8.8 million people (1.1–1.3%) are infected in our 22 focus countries. HCV-specific mortality, DALY, and transplantation data are unavailable. Estimations via HCV-attributable fractions indicate that HCV caused more than 86000 deaths and 1.2 million DALYs in the WHO European region in 2002. Most of the DALYs (95%) were accumulated by patients in preventable disease stages. About one-quarter of the liver transplants performed in 25 European countries in 2004 were attributable to HCV.

**Conclusion:**

Our results indicate that hepatitis C is a major health problem and highlight the importance of timely antiviral treatment. However, data on the burden of disease of hepatitis C in Europe are scarce, outdated or inconclusive, which indicates that hepatitis C is still a neglected disease in many countries. What is needed are public awareness, co-ordinated action plans, and better data. European physicians should be aware that many infections are still undetected, provide timely testing and antiviral treatment, and avoid iatrogenic transmission.

## Background

Since the discovery of the hepatitis C virus (HCV) in 1989 and its identification as one of the leading causes of chronic liver disease with life-threatening sequelae such as end-stage cirrhosis and liver cancer [1], questions have been raised about the burden of disease caused by the virus.

According to the World Health Organization (WHO), about 3% of the world's population is infected with HCV, with prevalence ranging from 0.1–5% in different European countries [2,3]. Approximately 15–25% of HCV infections are estimated to progress to severe liver disease, which may take more than 30 years to develop [1,4].

Acute infections and less-advanced stages of chronic disease usually are clinically silent [1,5], and only about half of the viremic patients exhibit elevated ALT activity [6]. Therefore, hepatitis C is often first diagnosed in a late stage when therapeutic options are already limited. Due to the slow and silent onset many patients are unaware of their infection. Even in France, which among European countries most actively screens for HCV-infection, at least 40% of the infections are still undetected [7].

Liver transplantation is the only therapeutic option for patients with end-stage liver disease [1,8]. If detected in time, however, progression to severe liver disease can be prevented in 54–63% (95% confidence limits ranging from 49–68%) of patients through antiviral treatment with peginterferon and ribavirin [9-13].

In many countries, transmission rates decreased substantially with the introduction of routine blood screening in 1991 [2,14,15]. However, due to slow disease progression, many patients infected prior to the 1990s via contaminated blood products are still at risk to progress to severe liver disease in future years. Therefore, despite the decline of new infections acquired via blood products, mathematical models still predict a continuing rise in HCV-related morbidity and mortality [16-21]. Today, after the eradication of transfusion-related infections, intravenous drug use is considered as the main cause of HCV transmission in most of European countries, with prevalence rates among intravenous drug users (IDUs) ranging from 15% to 90% [22-24]. In Eastern Europe, nosocomial infections seem to play an important role as well [22].

Burden of disease estimates are frequently cited in the literature. However, most estimates reflect provisional expert consensus opinion, and the empirical evidence underlying these estimates is seldom revealed. Experts have estimated that HCV accounts for 20% of cases of acute hepatitis, 70% of cases of chronic hepatitis, 40% of cases of end-stage cirrhosis, 60% of cases of hepatocellular carcinoma (HCC), and 30% of cases of liver transplants (LT) [3] in industrialised countries. According to the WHO, even two-thirds of liver transplants are due to HCV infection [25] and a US consensus conference named HCV as the primary reason for liver transplantation [26].

Motivated by the lack of reliable data needed to prioritise public health measures, an international working group was established to assist the WHO in estimating the global burden of disease associated with HCV infection [27]. However, important results from this working group are still preliminary or pending.

The objectives of this review were to: i) summarise burden of disease data presently available for countries of the WHO European region, including calculation of burden of disease estimates via attributable fractions where HCV-specific data are missing; ii) compare the data to current expert estimates; and iii) identify areas in which better data are needed. The burden of disease indicators we examined were incidence, prevalence, mortality, health-related quality of life, and liver transplantation.

## Methods

### Geographic Focus

Our review focused on the following 22 countries of the WHO European region [28]: Austria, Belgium, the Czech Republic, Denmark, Finland, France, Germany, Greece, Hungary, Ireland, Italy, the Netherlands, Norway, Poland, Portugal, Romania, Russia, Spain, Sweden, Switzerland, Turkey, and the United Kingdom. When the reviewed data sources yielded data for other countries of the region, these were reported as well.

### Literature and Data Search

Our primary goal was to retrieve data that are nationally representative and comparable across countries.

We performed a systematic literature search up to October 2006 in Medline, PreMedline, and Embase, combining search terms for HCV-related disease ("HCV" or "hepatitis C" or "cirrhosis" or "hepatocellular carcinoma" or "liver cancer"), with search terms for our predefined burden of disease indicators (incidence, prevalence, mortality, quality of life, liver transplantation) and geographic region (Europe, or one of our 22 focus countries). Because HCV was first discovered in 1989, we restricted our search to documents published since then. Documents in languages other than English or German, and studies on animals were excluded.

In addition, we reviewed reference lists of retrieved publications, searched websites of national and international organisations (e.g., Centers for Disease Control (CDC), Deutsche Stiftung Organtransplantation (DSO), European Association for the Study of the Liver (EASL), European Centre for Disease Prevention and Control (ECDC), European Liver Transplant Registry (ELTR), European Commission, Statistical Office of the European Communities (Eurostat), Eurosurveillance, National Institutes of Health (NIH), Organisation for Economic Co-operation and Development (OECD), World Health Organization (WHO) and consulted with experts from health organisations and pharmaceutical companies to obtain data from national sources. Consultation with experts was particularly helpful in the case of prevalence.

### Calculations and Reporting

In cases where aggregated burden of disease data were reported for multi-causal disease outcomes – such as liver cirrhosis or liver cancer – but HCV-specific data were missing, we used attributable fractions to estimate the number and proportion of cases related to HCV infection. Cases attributable to HCV were derived by weighting the total number of cases with respective HCV-attributable fractions. HCV-attributable fractions were derived from a recent publication by Perz et al. [29], who estimated the fractions of cirrhosis and hepatocellular carcinoma (HCC) attributable to hepatitis B virus and HCV infections in the WHO sub-regions. We preferred attributable fractions as derived by Perz et al. to those recently derived by other researchers [30] because those by Perz et al. do not rely on currently uncertain estimates of HCV prevalence. The use of regional, rather than country-specific, attributable fractions is a simplification that may not have a strong influence on regional burden of disease estimates, but can yield inaccurate results for individual countries.

To describe quality of life-related burden of disease, we used disability-adjusted life-years (DALYs) and years lost due to disability (YLDs) [31-36]. The DALY was introduced by the WHO Global Burden of Disease Study (GBD) to compare death and disability from various disorders across countries. It is a measure of population health that sums together years of life lost from premature death in the population (YLLs) and those lost due to being in a state of poor health or disability (YLDs) by incident cases. One DALY can be thought of as one year of 'healthy' life lost.

Retrieved and calculated burden of disease data are presented in detailed tables as additional material (see additional file 1). Evidence tables are presented for country-specific HCV incidence and prevalence, and HCV-related deaths, DALYs, and liver transplants. Input parameters and steps of our calculation are made transparent in the tables. In the main document, data are presented as choropleth maps of the WHO European region where the map categories represent rounded data quartiles. The maps were compiled in SAS (release 9.1 by SAS Institute Inc., Cary, NC, USA).

## Results

### Incidence

Our systematic literature search revealed a scarcity of HCV incidence data that are comparable across countries of the WHO European region. Pan-European HCV incidence data available from the WHO Health for All Database [37], which presents annual case numbers of acute hepatitis C (ICD-10 Code B17.1) submitted by countries of the WHO European region, proved to be the most comparable due to the use of a standardised case-definition.

Table 1 (see additional file 1) presents annual incidence rates of acute hepatitis C reported to the WHO from the European region. Data were available for the period 1997 to 2004. All countries except Monaco and Turkey provided at least one estimate, and only a few countries reported data on an annual basis. Incidence data for most countries varied with time. Data from some countries suggest a decrease in incidence, whereas other countries exhibit increases. In some countries incidence appears to rise and fall without a trend.

**Figure 1:**
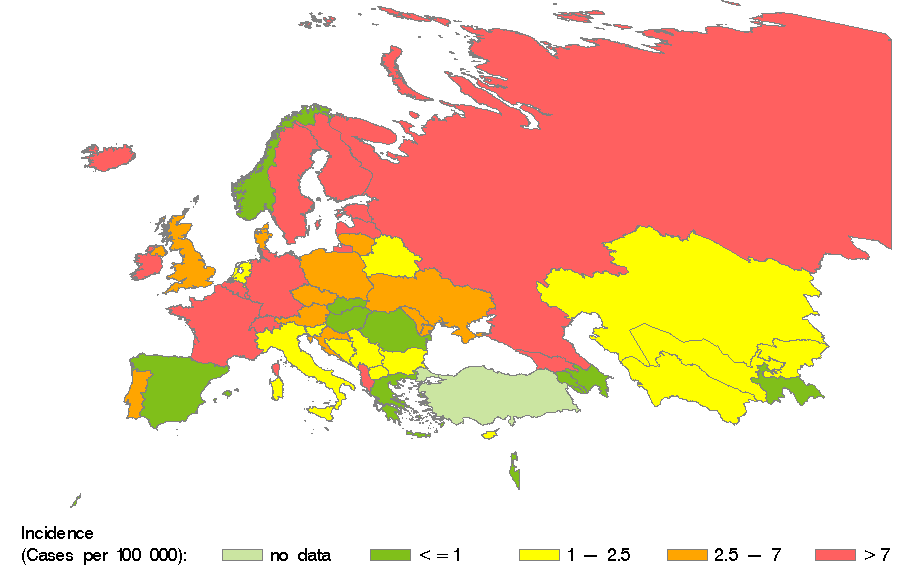
**Average annual incidence of HCV infection in countries of the WHO European region between 1997 and 2004. Source: Calculated from WHO Health for All data **[37]

To cope with temporal variation, we averaged annual incidence figures reported during the observed time period (see additional file 1, Table 1). Averaged annual incidence rates for acute hepatitis C vary across countries from 0.00 to 39.21 cases per 100000 residents. However, the geographic distribution of incidence rates in the WHO European region shows no clear pattern (Figure 1). Applying a population size weight yielded an average annual incidence rate of 6.19 per 100000 (95% CI 4.90–7.48) for the WHO European region (excluding Monaco and Turkey).

### Prevalence

Though our systematic literature search for prevalence data retrieved 335 references, almost all were considered inappropriate due to lacking representativeness for the general population. HCV prevalence data covering most European countries were available from the WHO, which published global HCV prevalence data submitted by countries or selected from published studies in 1997 and 1999 [38,39].

**Figure 2:**
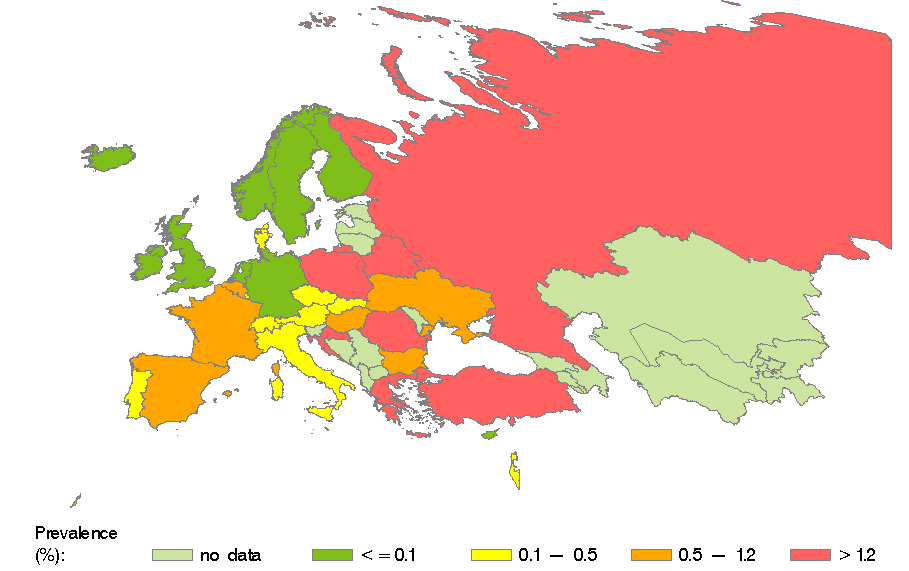
**Prevalence of HCV infection in countries of the WHO European region.** Source: WHO 1999 [39]

WHO prevalence data for the WHO European region are presented in Table 2 (see additional file 1). Estimates were provided for 32 countries of the region, including all 22 of our focus countries. Prevalence estimates range from 0.003% to 4.5%. The map compiled from the WHO data (Figure 2) indicates high prevalence rates (> 1.2%) in Southern and Eastern European countries, whereas low prevalence rates (<= 0.1%) predominate in Northern European countries.

We performed plausibility checks to evaluate the quality of WHO prevalence data. In a first plausibility check, we checked for consistency with data from other sources (see additional file 1, Table 2). Specifically, WHO figures were compared to data from a review of blood donor studies in seven European countries [40], and data from national sources. National data were retrieved from national health authorities or, if those were unavailable, from national key opinion leaders. National estimates could be retrieved for 21 of our 22 study countries, except Finland. Compared to these, WHO prevalence estimates are lower for 12 countries, about equal for six, and higher for three. Estimates from blood donor studies are substantially higher for five countries and lower for two.

In a second plausibility check, we investigated the consistency between WHO prevalence and incidence data that were available for 30 countries (see additional file 1, Tables 1 and 2). In order to screen for data inconsistencies, we divided prevalence by incidence, which, under steady-state assumptions, yields an estimate of disease duration (Duration ~Prevalence/Incidence) [41,42]. Calculated disease duration ranged from 0 to 23077 years. Even though the steady-state assumption does not hold, the extreme range indicates inconsistencies between WHO prevalence and incidence data. Inconsistencies are most likely in case of short disease duration, which indicates that high incidence is coupled with low prevalence. Prolonged disease duration does not necessarily reflect data inconsistencies but might come about in countries that have greatly reduced incidence through effective prevention programs.

According to WHO prevalence data, approximately 7.3 million (1.1%) people in our 22 focus countries are estimated to be infected with HCV. Based on the higher national estimates, 8.8 million (1.3%) people are estimated to be infected.

### Mortality

Our systematic literature search yielded little data on HCV-related mortality in the general population, since studies focused primarily on mortality in risk groups and patients co-infected with HIV. The most consistent country- and disease-specific mortality data were available from the 2002 WHO GBD [43], which were derived from vital registration statistics or other reliable sources [35]. Data for the countries of the WHO European region include the estimated number of deaths in 2002 attributable to hepatitis C infections without cirrhosis and liver cancer [44]. As only all-cause mortality data were reported for liver cancer and cirrhosis [44], deaths attributable to HCV for these conditions were calculated via attributable fractions. The total number of deaths attributed to HCV was calculated as the sum of deaths due to hepatitis C, HCV-related cirrhosis and HCV-related liver cancer.

**Figure 3:**
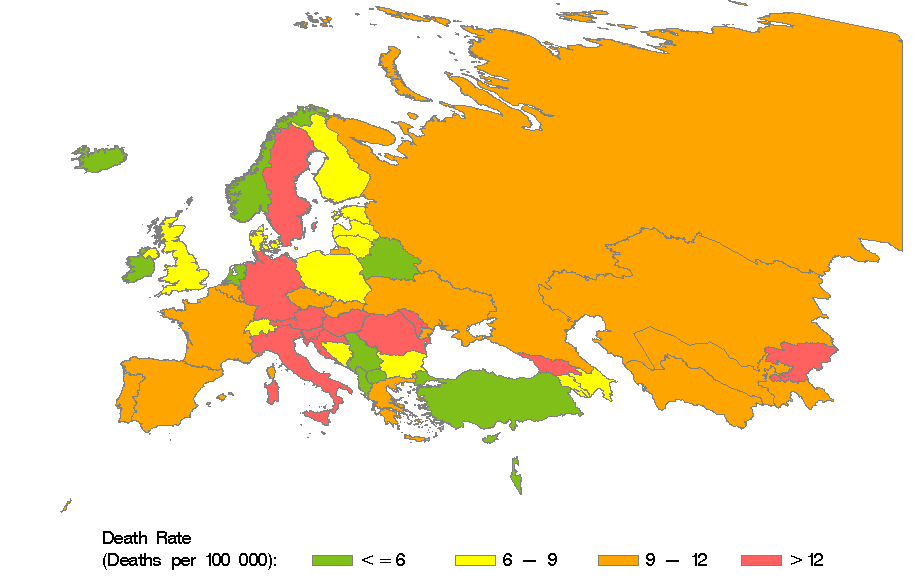
**HCV-related death rates in countries of the WHO European region in 2002.** Source: Calculated from WHO GBD data [44]

**Figure 4:**
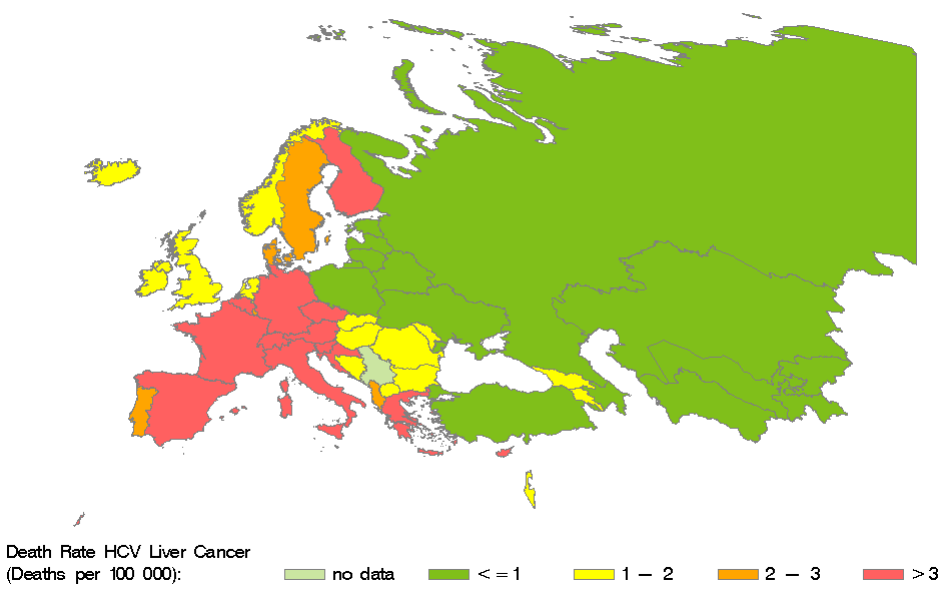
**Death rates for HCV-related liver cancer in countries of the WHO European region in 2002.** Source: Calculated from WHO GBD data [44]

**Figure 5:**
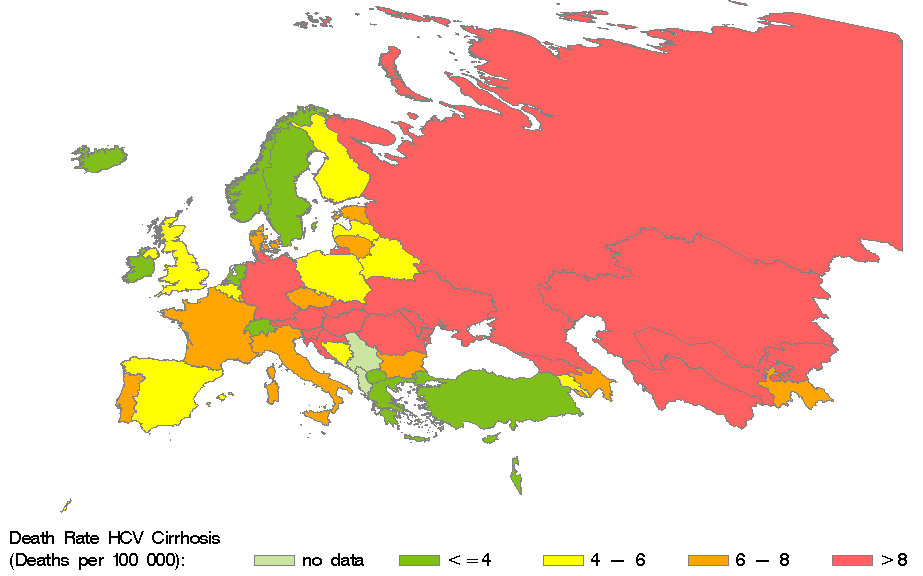
**Death rates for HCV-related liver cirrhosis in countries of the WHO European region in 2002.** Source: Calculated from WHO GBD data [44]

Retrieved and calculated mortality data are presented in Table 3 (see additional file 1). According to our calculations, HCV caused more than 86000 deaths in the WHO European region in 2002, accounting for 35% of cirrhosis and 32% of liver cancer deaths in that year. Country-specific HCV-related mortality ranges from 0.1 to 31.5 deaths per 100000 residents. Figure 3 shows the distribution of HCV-related death rates in the WHO European region. High death rates (> 12 deaths per 100000) are predominantly found in the centre of the region. HCV-related liver cancer mortality (Figure 4) displays a notable East-West gradient, with high death rates (> 3 per 100000) in Western Europe and low death rates (<= 1 per 100000) in Eastern Europe. The death rates for HCV-related cirrhosis (Figure 5) provide a complementary picture.

### Quality of life

Our systematic literature search produced several studies investigating health-related quality of life in patients with hepatitis C. However, only the WHO GBD reported burden of disease data that considered quality of life [43]. Available data comprised country-specific DALY [44] and region-specific YLD estimates [45] for the year 2002. As in the case of mortality, DALYs and YLDs were only reported for hepatitis C without cirrhosis and liver cancer [44]. Therefore, DALYs and YLDs resulting from HCV-related cirrhosis and liver cancer were again calculated via HCV-attributable fractions.

**Figure 6:**
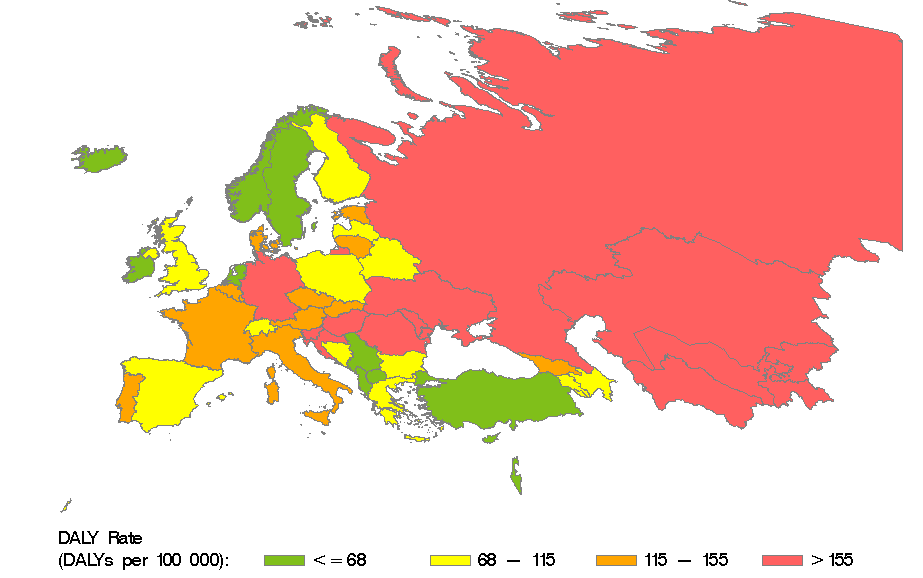
**HCV-related DALY rates in countries of the WHO European region in 2002.** Source: Calculated from WHO GBD data [44]

DALYs and DALY rates retrieved and calculated for the countries of the WHO European region are presented in Table 4 (see additional file 1). Based on our calculations, almost 1.2 million DALYs were lost due to HCV in the WHO European region in 2002, which corresponds to an overall rate of 134.54 DALYs per 100000 residents. Most DALYs (81%) were lost due to HCV-related cirrhosis. Figure 6 shows the distribution of HCV-related DALY rates in countries of the WHO European region. High rates (> 155 DALYs per 100000) predominate in Eastern countries.

We looked separately at YLDs, the disability component of the DALY measure. YLD data were available for the WHO European region as a whole but not for individual countries. HCV-related YLDs were calculated similarly to HCV-related DALYs by using the regional HCV-attributable fractions for cirrhosis (35%) and liver cancer (30%) derived from our DALY calculation. According to our calculations, HCV caused 200104 YLDs in the WHO European region in 2002. Of those YLDs, 6250 (3%) were due to hepatitis C without cirrhosis or liver cancer, 191537 (96%) were due to HCV-related cirrhosis and 2317 (1%) were due to HCV-related liver cancer.

### Liver transplantation

Because our systematic literature search did not yield Europe-wide HCV-specific data on liver transplants, these were calculated using data from various sources. We obtained the number of liver transplants performed in 2004 in 25 countries of the WHO European region, including 21 of our focus countries, from the Transplant Committee of the Council of Europe (TCCE) [46]; data were not available for the Russian Federation. Data on the distribution of indications for transplantation – including all-cause acute hepatic failure, cirrhosis, and liver cancer – were retrieved from the European Liver Transplant Registry (ELTR) [47]. HCV-attributable fractions were applied to obtain HCV-specific indication data. To derive numbers of HCV-related transplants, we weighted total numbers of liver transplants by all-cause indication and HCV-attributable fraction. Transplants due to virus-related acute hepatic failure were disregarded due to minor relevance and insufficient data.

Input and calculated data are presented in Table 5 (see additional file 1). According to the TCCE, 6411 liver transplants were performed in the 25 countries in 2004. Based on our calculations, 23% of those were attributable to HCV. HCV-related transplantation rates varied from 0.002 to 0.563 per 100000 residents (rates not shown in tables).

**Figure 7:**
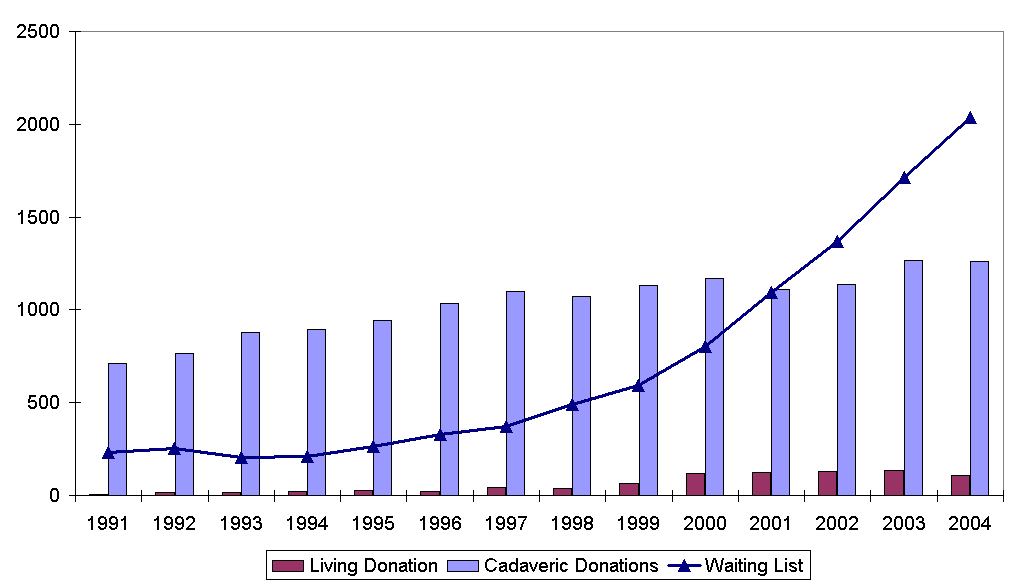
**Liver transplants and waiting list 1991 to 2004.** Source: Eurotransplant

Data illustrating the increasing shortage of donor organs for liver transplantation were retrieved from Eurotransplant, a mediation centre for organ donations active in seven European countries. As shown by Figure 7, the demand for liver transplantation (reflected by the waiting list) constantly increased over the last decade, whereas the number of transplantations (reflecting the organ supply) was stagnant.

## Discussion

Data on the burden of disease of hepatitis C in Europe are scarce, outdated or inconclusive. By revealing the paucity of available information, our study indicates that hepatitis C is still a neglected disease in many countries. The findings are relevant for physicians, researchers and health care decision makers concerned with hepatitis C virus infection in Europe.

Since our calculations are based on uncertain input data from various external sources and simplifying assumptions, our results are open to debate. A particular limitation of our analysis is the use of regional rather than country-specific HCV-attributable fractions. This may not have a strong influence on regional burden of disease estimates but can yield inaccurate results for individual countries. However, as attributable fractions are currently unavailable for many countries of the WHO European region, there was no alternative to using aggregated attributable fractions in order to estimate the HCV-related burden of disease for our selection of 22 countries or the WHO European region as a whole. Although, the quality of our input data strongly limits the inter-country comparability of our results, we present burden of disease estimates for individual countries in our tables. Since all parameters of our calculations have been made explicit, country-specific estimates can easily be revised by the interested reader as better data become available. Thus our results and tables could serve as a starting point for improved data collection and eventually, might contribute to move forward public health and patient care.

### Incidence

Incidence data are usually assessed by surveillance systems. However, although hepatitis C is a notifiable disease in most countries of the WHO European region, no uniform hepatitis C surveillance exists at the European level [48,49]. HCV incidence data provided by national surveillance are hardly comparable across countries due to different surveillance systems and case definitions [48-51]. Heterogeneous surveillance causes geographic variation of incidence estimates. Temporal variation might be due to changes in reporting behaviour, modifications of the surveillance system or case definitions, and increased sensitivity of tests (e.g., 2nd and 3rd generation anti-HCV assays). Variation and trends in incidence are therefore difficult to interpret [49].

A general problem for HCV surveillance is its poor sensitivity [3,5,51] resulting from under-detection and -reporting. Under-detection primarily results from the slow and silent onset of the disease [49,52]. Under-reporting has been described as a consequence of political unrest, disinterest, and poorly developed infrastructures [53,54]. Due to the insensitivity of hepatitis C surveillance, HCV attack-rates are systematically underestimated [55], especially if surveillance focuses only on acute infections which are rarely symptomatic. Targeted screening programs could improve the sensitivity of surveillance and reduce the number of undetected cases. Second-generation surveillance as proposed for HIV might help to understand and prevent the spread of the HCV epidemic [56].

Presently, the most comprehensive collection of European incidence data applying a uniform case definition is produced by the WHO [37]. Per definition, WHO data represent cases of acute hepatitis C, which in theory most closely approximate the incidence of HCV infections. However, since not all countries reporting to WHO in the past were able to distinguish acute from chronic cases [57], there is serious doubt that all cases truly meet the case definition. As newly diagnosed or reported cases of chronic hepatitis might have been misclassified as acute cases, WHO data do not necessarily reflect true incidence rates. Substantial variation of the reported incidence estimates suggest that the data are highly unreliable, which strongly impairs inter-country comparability as well. Based on WHO data, the population-weighted average annual incidence for acute hepatitis C in the WHO European region is 6.19 per 100000 residents (95% CI 4.90–7.48). Expert estimates and alternative data available for selected European countries are not always consistent with WHO incidence data [3,48,58]. However, considering the general uncertainty of HCV incidence data, it is difficult to decide which data are most valid.

In general, incidence data focussing on acute infections do not appropriately reflect the size of the hepatitis C problem because many infections were acquired through contaminated blood products prior to the 1990s. Therefore, even in countries with presently low transmission rates, the prevalence of hepatitis C might be high.

### Prevalence

Given that many infections were contracted in the past, HCV prevalence is the key measure in quantifying the size of the hepatitis C problem and guiding health care resource planning. In contrast to incidence data which are collected by surveillance systems, the assessment of prevalence requires representative population surveys. Even studies assessing the prevalence in blood donors are problematic, since high-risk groups such as intravenous drug users are often excluded from blood donation, leading to an underestimation of the true prevalence in the population [59]. HCV prevalence assessed in population surveys can easily be underestimated as well, if there is selection bias regarding high risk groups like IDUs [60,61]. Therefore, some countries have corrected prevalence estimates for selection bias.

Our investigation suggests that, the WHO should currently be considered as the most comprehensive source of European HCV prevalence data because their data were collected by a sole authority with uniform demands on data quality, and comparable data collections are lacking. Nevertheless, it is widely accepted that current data do not necessarily represent true HCV prevalence and are in need of updating [27,29,39,59]. Additionally, the lack of age-specific prevalence data needed for burden-of-disease projections has been criticised.

As revealed by our investigation, 1999 WHO prevalence data frequently differ from estimates from other sources and are not always consistent with incidence data, underscoring the uncertain data quality. While identifying which specific prevalence data are incorrect was beyond the scope of our study, our investigation indicates that WHO data for Europe tend to be lower than prevalence data from other sources. That is, estimates communicated by experts in the field [62,63], results from prevalence studies published after 1999 [6,64-68], and most interestingly, data communicated on national levels, are all often higher than WHO estimates. This finding is important because WHO data are disseminated on an international level and used widely in research and health policy, as demonstrated by several recent publications referring to the 1999 WHO data [14,30,51,59]. The reasons for which WHO prevalence data for most countries deviate from national estimates are unclear. While it is possible that national information is not effectively passed on to the international level, concerns about data validity and/or comparability must be considered as well. Evaluating the quality of national prevalence estimates is difficult because those estimates are often reported only in context documents without clear description of the underlying data source(s). An explanation for this may be that national prevalence estimates are based on a variety of study results and modelling assumptions, when representative population surveys are lacking or correction for selection bias is needed.

Applying WHO and national prevalence data, we estimated that 1.1% to 1.3% of the population in our 22 focus countries are infected with HCV. However, as our selection of countries is arbitrary, this prevalence may not be applicable for the WHO European region as a whole. Further studies assessing the age-specific HCV prevalence in representative population samples are needed, and data disseminated on the international level should be updated.

### Mortality

According to our investigation, HCV-specific mortality in Europe has not yet been sufficiently assessed. According to our calculations, HCV caused more than 86000 deaths in the WHO European region in 2002. This is about the same number of deaths attributed to pancreas cancer (ICD-10 Code C25) and more than twice the number estimated for HIV/AIDS (ICD-10 Code B20-B24) [69].

HCV was estimated to account for 35% of cirrhosis and 32% of liver cancer deaths, respectively. The latter is not consistent with the frequently cited statement that HCV accounts for 60% of hepatocellular carcinomas [3,25]. Since HCC is the most frequent type of liver cancer [70] and survival with liver cancer is short [71], the proportion of liver cancer deaths due to HCV should approximate the HCV-attributable fraction for liver cancers, which in our analysis ranged from 15 to 44%. As these fractions represent regional averages, our estimate of HCV-related liver cancer deaths might be inaccurate for individual countries. In Italy, for example, study data suggest higher HCV-attributable fractions between 50% and 70% [72-74].

Our data show opposite East-West gradients for HCV-related cirrhosis and liver cancer mortality, with lower HCV-related cancer mortality in the East. This might be the result of a competing risk situation in which patients in the East die from cirrhosis before developing liver cancer. HCV-related cirrhosis mortality might be higher due to concomitant excess alcohol consumption [75]. An alternative explanation would be that HCC in Eastern Europe is under-detected or misclassified as cirrhosis, which is likely because state-of-the-art imaging technology is less frequently available.

In general, geographic variation of HCV-related mortality rates may be caused by differences in HCV prevalence. However, heterogeneous data quality, deviating health care standards, or differences in the distribution of competing and synergistic risk factors should be considered as alternative explanations. For example, HCV-related death rates might be lower in countries with high prevalence of hepatitis B [2] and higher in countries with a high level of alcohol consumption [75]. Specifically, heavy alcohol consumption has been shown to be associated with higher risks of cirrhosis, liver cancer and death in patients with chronic hepatitis C [29,76].

Generally, our mortality data represent crude estimates that are unadjusted for potential co-factors. Therefore, discussion of HCV-related deaths does not exclude the involvement of other risk factors or co-disease.

### Quality of life

Presently, the only data allowing a consistent cross-country comparison of burden of disease resulting from HCV-related quality of life impairment are DALYs and YLDs estimated by the WHO GBD in 2002. No expert estimates are available for such a burden. HCV-related DALYs and YLDs reported by the GBD are low due the exclusion of cases with cirrhosis and liver cancer. When these cases are included – as in our analysis – the picture changes dramatically. According to our calculations, HCV caused approximately 1.2 million DALYs in the WHO European region in 2002, of which about one sixth (200104 YLDs) are attributed to quality of life impairment. Comparing this figure to DALYs reported by the WHO GBD for other diseases reveals that hepatitis C is a major health problem, which is almost as big as HIV/AIDS (ICD-10 Code B20-B24) or stomach cancer (ICD-10 Code C16), each causing about 1.4 million DALYS in 2002 [77].

High DALY rates are predominantly found in Eastern European countries where HCV-related cirrhosis mortality is high but HCV-related liver cancer mortality is low. This finding can be partially explained by the nature of the DALY measure itself which puts strong weight on life-years lost due to premature death. Thus, our results imply that hepatitis C patients in Eastern European countries die at a younger age, on average, than their Western European counterparts.

Roughly 95% of the HCV-related DALYs were accumulated by patients in advanced disease stages (cirrhosis or liver cancer). This figure highlights the importance and potential benefit of antiviral treatment, which can prevent disease progression and advanced liver disease. This result seems surprising, as several studies have shown that HCV infection diminishes health-related quality of life even in the absence of advanced liver disease [78-84] and there are more patients with mild than with severe disease. Our data suggest that quality-of-life impairment caused by HCV infection is less relevant from a burden of disease perspective, because (1) the DALY measure gives a strong weight on life-years lost due to premature death, (2) disability weights applied by the Global Burden of Disease study for advanced stages – consistently with published patient-derived quality-of-life indices (utilities) [85,86] – were 3 to 11 times higher then those applied for early stages, and most importantly, (3) YLDs, the disability component of the DALY measure, focus only on incident cases. However, uncertainty remains, as it is unclear which conditions and aspects are covered by the disability weight of 0.075 [35] that was used for the calculation of hepatitis C-related DALYs in the GBD and the estimation of YLDs (and, by extension, DALYs) required a broad set of input data from a multitude of sources [87].

### Liver transplantation

Due to the lack of comprehensive data, HCV-related liver transplantations in Europe were calculated using input data from various sources. According to our calculations, about one-quarter of liver transplants in Europe are related to HCV.

Although this figure is lower than previous expert estimates [3,88], under-estimation from our data seems unlikely considering that our calculations are based on the assumption that 34% to 38% of cirrhosis-related transplants are due to HCV, while ELTR reported a much lower fraction of 16% [47]. However, results of our calculations are limited by the use of uncertain input data and simplifying assumptions. Additionally, our estimates might not be valid for individual countries due to the use of regionally aggregated HCV-attributable fractions.

Even with an attributable fraction of 23%, HCV is a dominant reason for liver transplantation. Considering the already-existing shortage of donor organs [47,89] and that many HCV infections acquired prior to the 1990s have not yet progressed to the stage of cirrhosis [90], the need for health care action, such as timely antiviral treatment of hepatitis C patients, is obvious. As suggested by models projecting the incidence of HCV-related cirrhosis and liver cancer, the need for liver transplantation will continue to rise considerably over the next 10 to 20 years without treatment [16-19].

According to our data, HCV-related transplantation rates in the European region vary considerably independent of HCV prevalence. This might indicate asymmetric reporting quality or unequal access to liver transplantation.

As shown by our review, European data on HCV-related liver transplants, like HCV-related burden of disease data in general, are poor. However, while the improvement of incidence, prevalence, mortality and DALY data requires considerable efforts, reliable HCV-related transplant data could easily be generated by collecting little additional HCV-specific information. This task could either be adopted by the Transplant Committee of the Council of Europe (TCCE) or the European Liver Transplant Registry (ELTR).

## Conclusion

Our results indicate that hepatitis C is a major health problem and highlight the importance of timely antiviral treatment. However, data on the burden of disease of hepatitis C in Europe are scarce, outdated or inconclusive. By revealing the paucity of available information, our study indicates that hepatitis C is still a neglected disease in many countries. What is needed are public awareness, co-ordinated action plans, and better data. As the implementation of national action plans will take time, awareness among physicians is crucial. Specifically, physicians should be aware that many infections are still undetected, provide timely testing and antiviral treatment, and avoid iatrogenic transmission. Our results could serve as a foundation for improved data collection in the future.

## Competing interests

This project was supported in part by an unrestricted educational grant from Hoffmann La-Roche Ltd., Basel, Switzerland. The authors had complete and independent control over study design, analysis and interpretation of data, report writing, and publication, regardless of results. Drs. Mühlberger and Siebert received speakers honorarium and/or travel support from Hoffmann La-Roche Ltd. to present preliminary results of the study. Dr. Siebert and G. Sroczynski received funding from different health technology assessment agencies to perform health technology assessments related to hepatitis C (Canadian Agency for Drugs and Technologies in Health, German Agency for Health Technology Assessment at the German Institute for Medical Information and Documentation/German Federal Ministry of Health, Institute for Technology Assessment at the Austrian Academy of Sciences, Austrian Ludwig Boltzmann Institute for Health Technology Assessment) and unrestricted research grants from Roche and Schering Plough to perform cost-effectiveness studies for hepatitis C. Dr. Zeuzem received honoraria for lectures and advisory board meetings and served as clinical investigator for Roche and Schering Plough.

## Authors' contributions

The review was designed and co-ordinated by NM, US and SZ. RS, NM, BL and GS were involved in literature search, data acquisition and data extraction. Data analyses were done by NM and US. All authors contributed to result interpretation, were actively involved in the writing of the manuscript, and approved the version submitted for publication.

## Pre-publication history

The pre-publication history for this paper can be accessed here:



## Supplementary Material

Additional file 1 **Tables 1 to 5.** Table 1: Incidence – Cases of acute hepatitis C per 100,000 inhabitants reported to WHO by countries of the WHO European region between 1997 and 2004. Table 2: Prevalence – Hepatitis C prevalence in countries of the WHO European region reported by WHO, the Hepatitis C European Network for Co-operative Research (HENCOR) and national sources. Table 3: Mortality – Deaths and death rates related to HCV in countries of the WHO European region in 2002. Table 4: DALYs and DALY rates related to HCV in countries of the WHO European region in 2002. Table 5: Transplantation – Number and proportion of liver transplants (LT) due to HCV related cirrhosis and liver cancer (LC) in countries of the WHO European region in 2004 Click here for file
