# Supplementary material for: HCV-related burden of disease in Europe: a systematic assessment of incidence, prevalence, morbidity, and mortality
Source: BMC Public Health. 2009 Jan 22;9:34. doi: 10.1186/1471-2458-9-34 (PMC2656539; doi:10.1186/1471-2458-9-34)
Supplement: Additional file 1 — Tables 1 to 5. Table 1: Incidence – Cases of acute hepatitis C per 100,000 inhabitants reported to WHO by countries of the WHO European region between 1997 and 2004. Table 2: Prevalence – Hepatitis C prevalence in countries of the WHO European region reported by WHO, the Hepatitis C European Network for Co-operative Research (HENCOR) and national sources. Table 3: Mortality – Deaths and death rates related to HCV in countries of the WHO European region in 2002. Table 4: DALYs and DALY rates related to HCV in countries of the WHO European region in 2002. Table 5: Transplantation – Number and proportion of liver transplants (LT) due to HCV related cirrhosis and liver cancer (LC) in countries of the WHO European region in 2004 [file 1471-2458-9-34-S1.doc]

Table 1: Incidence - Cases of acute hepatitis C per 100,000 inhabitants reported to WHO by countries of the WHO European region between 1997 and 2004

| **Countries of the** | **Population ^a^** | **Annual cases of acute hepatitis C per 100,000 ^b^** | | | | | | | | **Average** |
| --- | --- | --- | --- | --- | --- | --- | --- | --- | --- | --- |
| **WHO European Region** |  | **1997** | **1998** | **1999** | **2000** | **2001** | **2002** | **2003** | **2004** | **1997-2004** |
|  | **('000)** |  |  |  |  |  |  |  |  | **(95% CI)** |
| Albania | *3,141* |  |  | *22.02* | *33.46* | *34.57* |  |  |  | 30.02 (12.76-47.28) |
| Andorra | *69* |  |  | *6.07* | *7.59* | *0* | *0* | *1.43* | *1.34* | 2.74 (0.00-6.16) |
| Armenia | *3,072* | *0* |  | *0.15* |  |  |  |  |  | 0.08 (0.00-1.03) |
| Austria | *8,111* | *3.89* | *4.73* | *7.02* | *5.08* | *4.32* | *7.23* | *13.14* |  | 6.49 (3.52-9.45) |
| Azerbaijan | *8,297* |  |  |  |  | *0.2* | *0.23* | *0.58* |  | 0.34 (0.00-0.86) |
| Belarus | *9,940* | *2.4* | *1.92* | *1.87* | *2.53* | *2.67* | *1.81* | *1.22* | *1.26* | 1.96 (1.50-2.42) |
| Belgium | *10,296* | *1.67* |  |  | *4.21* | *8.25* | *9.18* | *11.92* | *6.99* | 7.04 (3.20-10.87) |
| Bosnia and Herzegovina | *4,126* | *1* | *1.04* | *1.98* | *1.11* | *1.86* | *2.23* | *3.75* | *2.38* | 1.92 (1.15-2.69) |
| Bulgaria | *7,965* | *0.52* | *0.8* | *0.97* | *1.08* | *1.3* | *1.66* | *1.85* | *1.88* | 1.26 (0.84-1.68) |
| Croatia | *4,439* | *4.18* | *3.67* | *3.65* | *3.38* | *2.88* | *4.95* | *5.02* | *5.65* | 4.17 (3.38-4.97) |
| Cyprus | *796* |  |  |  |  |  |  | *1.25* | *0.95* | 1.10 (0.00-3.01) |
| Czech Republic | *10,246* | *2.18* | *3.1* | *3.2* | *3.11* | *2.7* | *2.09* | *1.78* | *1.93* | 2.51 (2.02-3.00) |
| **Denmark** | *5,351* | *2.74* | *3.62* | *2.29* | *5.45* | *5.21* | *5.08* | *4.33* |  | 4.10 (2.94-5.26) |
| Estonia | *1,338* | *19.44* | *26.4* | *17.74* | *26.65* | *22.43* | *14.65* | *11.38* | *9.19* | 18.49 (13.03-23.94) |
| Finland | *5,197* | *37.04* | *34.99* | *33.94* | *33.6* | *28.74* | *26.34* | *24.21* | *23.56* | 30.30 (25.92-34.68) |
| **France** | *59,850* | *9.9* |  |  |  |  |  |  |  | 9.90 ( - ) |
| Georgia | *5,177* | *1.91* | *4.87* | *22.47* | *10.87* | *12.47* | *13.01* | *11.78* | *11.64* | 11.13 (6.04-16.21) |
| Germany | *82,414* | *7.07* |  |  |  | *10.57* | *8.18* | *8.44* |  | 8.57 (6.24-10.89) |
| **Greece** | *10,970* | *0.29* | *1.08* | *1.49* | *1.4* | *1.11* | *0.63* | *0.52* | *0.24* | 0.85 (0.44-1.25) |
| **Hungary** | *9,923* |  | *0.8* |  |  | *0.42* | *0.41* | *0.3* | *0.4* | 0.47 (0.23-0.71) |
| Iceland | *287* | *19.56* | *24.11* | *30.3* | *30.59* | *27.36* | *23.3* | *24.54* | *21.19* | 25.12 (21.76-28.47) |
| Ireland | *3,911* |  |  |  |  | *1.72* |  |  | *22.73* | 12.23 (0.00-145.70) |
| Israel | *6,304* | *1.53* | *0.5* | *0.78* | *0.7* | *0.75* | *0.55* | *1.52* | *0.63* | 0.87 (0.52-1.22) |
| Italy | *57,482* | *1.64* | *1.48* | *1.38* | *0.39* | *0.88* | *0.71* | *0.7* |  | 1.03 (0.59-1.46) |
| Kazakhstan | *15,469* |  | *1.13* | *1.53* | *2.82* |  | *1.48* | *1.48* |  | 1.69 (0.88-2.50) |
| Kyrgyzstan | *5,067* | *1.32* | *1.2* | *0.81* | *1.74* | *1.85* |  | *2.24* | *2.39* | 1.65 (1.12-2.18) |
| Latvia | *2,329* | *4.19* | *6.97* | *10.33* | *12.52* | *8.7* | *6.37* | *5.2* | *4.89* | 7.40 (4.97-9.83) |
| Lithuania | *3,465* | *3.13* | *3.13* | *3.4* | *3* | *5.69* | *3.69* | *2.81* | *2.42* | 3.41 (2.58-4.24) |
| Luxembourg | *447* | *15.91* | *13.37* |  | *12.77* |  |  | *2* | *0.44* | 8.90 (0.05-17.75) |
| Malta | *393* | *1.87* | *0.53* | *0.53* | *0* | *0* | *0.25* | *0* | *0.5* | 0.46 (0.00-0.98) |
| Monaco | *34* |  |  |  |  |  |  |  |  |  |
| Netherlands | *16,067* | *0* | *0* | *1.57* | *3.18* | *3.48* | *3.38* | *1.79* | *0.22* | 1.70 (0.43-2.97) |
| **Norway** | *4,514* | *0.48* | *0.54* | *0.56* | *0.49* | *0.78* | *0.53* | *0.79* | *0.78* | 0.62 (0.50-0.73) |
| **Poland** | *38,622* | *2.75* | *4.42* | *5.17* | *5.4* | *5.05* | *5.17* | *5.9* | *5.64* | 4.94 (4.11-5.76) |
| **Portugal** | *10,049* | *4.76* | *6.88* | *4.04* | *1.99* | *2.44* | *1.98* | *0.74* |  | 3.26 (1.33-5.20) |
| Moldova | *4,270* | *6.17* | *6.14* | *4.36* |  |  |  |  |  | 5.56 (2.98-8.13) |
| Romania | *22,387* |  |  | *0.08* | *0.12* | *0.18* |  | *0.4* |  | 0.20 (0.00-0.42) |
| **Russian Federation** | *144,082* | *9.19* | *11.65* | *21.12* | *0.19* | *16.83* |  |  |  | 11.80 (1.91-21.69) |
| San Marino | *27* | *0* |  | *3.76* | *0* |  |  |  |  | 1.25 (0.00-6.65) |
| Serbia and Montenegro | *10,535* | *1.66* |  |  | *1.36* | *1.62* |  |  | *1.36* | 1.50 (1.24-1.76) |
| Slovakia | *5,398* | *0.71* | *0.76* | *0.65* | *0.89* | *1.34* | *0.86* | *0.7* | *0.37* | 0.79 (0.56-1.01) |
| Slovenia | *1,986* | *2.37* | *2.62* | *2.32* | *2.61* | *0.5* | *0.35* | *0.55* | *0.65* | 1.50 (0.61-2.38) |
| Spain | *40,977* |  |  |  |  |  | *0* |  | *1.61* | 0.81 (0.00-11.03) |
| **Sweden** | *8,867* | *48.01* | *41.12* | *39.48* | *38.77* | *39.32* | *37.89* | *35.97* | *33.12* | 39.21 (35.60-42.82) |
| **Switzerland** | *7,171* | *39.23* | *39.79* | *40.23* | *33.56* | *32.03* | *35.36* | *1.19* | *1.06* | 27.81 (13.82-41.80) |
| Tajikistan | *6,195* |  |  |  |  | *0* |  |  |  | 0.00 (.-.) |
| Macedonia | *2,046* | *1.2* | *0.85* | *0.99* | *1.28* | *0.98* | *1.73* | *1.63* | *2.02* | 1.34 (0.99-1.68) |
| Turkey | *70,318* |  |  |  |  |  |  |  |  |  |
| Turkmenistan | *4,794* |  |  |  |  | *1.1* |  |  |  | 1.10 (.-.) |
| Ukraine | *48,902* |  |  |  |  |  |  | *2.79* |  | 2.79 (.-.) |
| United Kingdom | *59,068* | *0.57* | *1.05* | *1.27* | *1.77* | *1.79* | *2.26* | *2.64* | *12.52* | 2.98 (0.00-6.25) |
| Uzbekistan | *25,705* | *0.09* | *0.56* | *2.2* |  | *1.79* |  |  |  | 1.16 (0.00-2.75) |
|  |  |  |  |  |  |  |  |  |  |  |
| ALL |  |  |  |  |  |  |  |  |  | **6.19 (4.90-7.48)** |

Annual average incidence rates for single countries represent the sum of annual incidence rates divided by the number of years with available data. County-specific 95% confidence intervals (95% CI) represent partly truncated confidence intervals for the mean incidences. Overall average and 95% confidence interval are derived from weighted and stratified analysis accounting for intra- and inter-country variation and different population sizes of countries.
Figures presented in italics represent external input data; figures in standard font indicate calculated results. Country names in bold indicate the 22 focus countries of our report.
Data sources:
a) WHO GBD data 2002 available at: http://www3.who.int/whosis/burden/estimates/2002Rev/2002RevCountries/DthDALY2002.zip
b) WHO Health for All Database available at: <http://data.euro.who.int/hfadb/> (Note: Due to potential misclassification and insensitivity of hepatitis C surveillance, WHO data do not necessarily reflect true incidence rates - see discussion)

Table 2: Prevalence – Hepatitis C prevalence in countries of the WHO European region reported by WHO, the Hepatitis C European Network for Co-operative Research (HENCOR) and national sources

| **Countries of** | **Population ^a^** | **HCV-Prevalence (%)** | | |
| --- | --- | --- | --- | --- |
| **WHO European Region** |  | **WHO** | **HENCOR** | **National Sources** |
|  | **('000)** | **1999 ^b^** | **2000 ^c^** | **2006 ^d^** |
| Albania | *3,141* |  |  |  |
| Andorra | *69* |  |  |  |
| Armenia | *3,072* |  |  |  |
| Austria | *8,111* | 0.2 |  | 0.5 – 1* |
| Azerbaijan | *8,297* |  |  |  |
| Belarus | *9,940* | 1.4 |  |  |
| Belgium | *10,296* | 0.9 |  | 1 |
| Bosnia and Herzegovina | *4,126* |  |  |  |
| Bulgaria | *7,965* | 1.1 |  |  |
| Croatia | *4,439* | 1.4 |  |  |
| Cyprus | *796* | 0.1 |  |  |
| Czech Republic | *10,246* | 0.2 |  | 0.2 |
| **Denmark** | *5,351* | 0.2 |  | 0.7* |
| Estonia | *1,338* |  |  |  |
| Finland | *5,197* | 0.02 |  |  |
| **France** | *59,850* | 1.1 | 0.13 | 0.84 |
| Georgia | *5,177* |  |  |  |
| Germany | *82,414* | 0.1 | 0.22 | 0.4 - 0.7 |
| **Greece** | *10,970* | 1.5 | 0.56 | 1 - 1.1* |
| **Hungary** | *9,923* | 0.9 |  | 0.7 - 0.9* |
| Iceland | *287* | 0.1 |  |  |
| Ireland | *3,911* | 0.1 |  | 0.01 - 1.4 |
| Israel | *6,304* | 0.4 |  |  |
| Italy | *57,482* | 0.5 | 1.71 | 3 |
| Kazakhstan | *15,469* |  |  |  |
| Kyrgyzstan | *5,067* |  |  |  |
| Latvia | *2,329* |  |  |  |
| Lithuania | *3,465* |  |  |  |
| Luxembourg | *447* | 0.5 |  |  |
| Malta | *393* |  |  |  |
| Monaco | *34* |  |  |  |
| Netherlands | *16,067* | 0.1 |  | 0.1 - 0.4 |
| **Norway** | *4,514* | 0.1 |  | 0.5 - 0.7 |
| **Poland** | *38,622* | 1.4 |  | 1.4 - 1.6* |
| **Portugal** | *10,049* | 0.5 |  | 1.3* |
| Moldova | *4,270* |  |  |  |
| Romania | *22,387* | 4.5 |  | 4.5 |
| **Russian Federation** | *144,082* | 2.0 |  | 0.9 – 2* |
| San Marino | *27* |  |  |  |
| Serbia and Montenegro | *10,535* |  |  |  |
| Slovakia | *5,398* | 0.4 |  |  |
| Slovenia | *1,986* |  |  |  |
| Spain | *40,977* | 0.7 | 1.80 | 1 - 2 |
| **Sweden** | *8,867* | 0.003 | 0.24 | 0.5 |
| **Switzerland** | *7,171* | 0.2 |  | 0.5 - 1 |
| Tajikistan | *6,195* |  |  |  |
| Macedonia | *2,046* |  |  |  |
| Turkey | *70,318* | 1.5 |  | 0.8 - 1.2* |
| Turkmenistan | *4,794* |  |  |  |
| Ukraine | *48,902* | 1.2 |  |  |
| United Kingdom | *59,068* | 0.02 | 0.04 | 0.4 - 0.7 |
| Uzbekistan | *25,705* |  |  |  |

Country names in bold indicate the 22 focus countries of our report.
Data sources:
a) WHO GBD data 2002 available at: http://www3.who.int/whosis/burden/estimates/2002Rev/2002RevCountries/DthDALY2002.zip
b) WHO: Weekly Epidemiological Record. N° 49, 10 December 1999, WHO (http://www.who.int/docstore/wer/pdf/1999/wer7449.pdf)
c) HENCOR: Touzet S, et al. Epidemiology of hepatitis C virus infection in seven European Union countries: a critical analysis of the literature. HENCORE Group. (Hepatitis C European Network for Co-operative Research. European Journal of Gastroenterology & Hepatology 2000;12(6):667-78.
d) National sources: National health authorities or key opinion leaders. Stars (*) indicate that estimates were derived from key opinion leaders because no estimates were available from official national sources.

Table 3: Mortality - Deaths and death rates related to HCV in countries of the WHO European region in 2002

|  |  | **HEPATITIS** |  | **CIRRHOSIS** | | | |  | **LIVER CANCER** | | | |  | **ALL** | |
| --- | --- | --- | --- | --- | --- | --- | --- | --- | --- | --- | --- | --- | --- | --- | --- |
| **Countries of the** | **Population** | **Deaths** |  | **Total** |  | **Deaths** | |  | **Total** |  | **Deaths** | |  | **Deaths** | |
| **WHO European Region** |  | **attr. to HCV** |  | **Deaths** | **AF** | **attr. to HCV** | |  | **Deaths** | **AF** | **attr. to HCV** | |  | **attr. to HCV** | |
|  | **('000)** | **n** |  | **n** | **%** | **n** | **per 100,000** |  | **n** | **%** | **n** | **per 100,000** |  | **n** | **per 100,000** |
| Albania | *3,141* | *4* |  |  | *34* |  |  |  | *552* | *15* | 83 | 2.6384 |  | 87 | 2.7585 |
| Andorra | *69* | *1* |  | *9* | *38* | 4 | 5.1058 |  | *8* | *44* | 3 | 4.7749 |  | 8 | 11.1412 |
| Armenia | *3,072* | *1* |  | *496* | *34* | 169 | 5.4893 |  | *262* | *15* | 39 | 1.2809 |  | 209 | 6.8117 |
| **Austria** | *8,111* | *36* |  | *1758* | *38* | 668 | 8.2355 |  | *712* | *44* | 313 | 3.8619 |  | 1017 | 12.5441 |
| Azerbaijan | *8,297* | *18* |  | *1532* | *34* | 521 | 6.2775 |  | *517* | *15* | 78 | 0.9352 |  | 616 | 7.4289 |
| Belarus | *9,940* | *4* |  | *1538* | *34* | 523 | 5.2597 |  | *439* | *15* | 66 | 0.6620 |  | 592 | 5.9593 |
| **Belgium** | *10,296* | *74* |  | *1622* | *38* | 616 | 5.9860 |  | *704* | *44* | 310 | 3.0088 |  | 1000 | 9.7147 |
| Bosnia and Herzegovina | *4,126* | *6* |  | *517* | *34* | 176 | 4.2602 |  | *545* | *15* | 82 | 1.9831 |  | 263 | 6.3806 |
| Bulgaria | *7,965* | *13* |  | *1494* | *34* | 508 | 6.3788 |  | *802* | *15* | 120 | 1.5097 |  | 642 | 8.0574 |
| Croatia | *4,439* | *5* |  | *1392* | *38* | 529 | 11.9201 |  | *417* | *44* | 183 | 4.1337 |  | 718 | 16.1765 |
| Cyprus | *796* |  |  | *35* | *38* | 13 | 1.6768 |  | *61* | *44* | 27 | 3.3731 |  | 40 | 5.0500 |
| **Czech Republic** | *10,246* | *5* |  | *1812* | *38* | 689 | 6.7202 |  | *943* | *44* | 415 | 4.0502 |  | 1109 | 10.8193 |
| **Denmark** | *5,351* | *5* |  | *889* | *38* | 338 | 6.3126 |  | *268* | *44* | 118 | 2.2023 |  | 460 | 8.6002 |
| Estonia | *1,338* | *1* |  | *309* | *34* | 105 | 7.8524 |  | *79* | *15* | 12 | 0.8810 |  | 118 | 8.7979 |
| **Finland** | *5,197* | *9* |  | *729* | *38* | 277 | 5.3323 |  | *387* | *44* | 170 | 3.2757 |  | 457 | 8.7847 |
| **France** | *59,850* | *147* |  | *9475* | *38* | 3600 | 6.0157 |  | *7129* | *44* | 3137 | 5.2409 |  | 6884 | 11.5015 |
| Georgia | *5,177* | *0* |  | *1641* | *34* | 558 | 10.7772 |  | *434* | *15* | 65 | 1.2560 |  | 623 | 12.0395 |
| **Germany** | *82,414* | *809* |  | *17979* | *38* | 6832 | 8.2897 |  | *6217* | *44* | 2735 | 3.3190 |  | 10376 | 12.5903 |
| **Greece** | *10,970* | *35* |  | *714* | *38* | 271 | 2.4726 |  | *2038* | *44* | 897 | 8.1758 |  | 1204 | 10.9715 |
| **Hungary** | *9,923* | *14* |  | *5652* | *34* | 1922 | 19.3660 |  | *863* | *15* | 129 | 1.3050 |  | 2065 | 20.8076 |
| Iceland | *287* | *0* |  | *6* | *38* | 2 | 0.7293 |  | *12* | *44* | 5 | 1.8130 |  | 8 | 2.6501 |
| **Ireland** | *3,911* | *9* |  | *156* | *38* | 59 | 1.5167 |  | *169* | *44* | 74 | 1.9005 |  | 143 | 3.6581 |
| Israel | *6,304* | *76* |  | *257* | *38* | 98 | 1.5477 |  | *193* | *44* | 85 | 1.3462 |  | 259 | 4.1044 |
| **Italy** | *57,482* | *1208* |  | *11035* | *38* | 4193 | 7.2947 |  | *10533* | *44* | 4635 | 8.0627 |  | 10036 | 17.4589 |
| Kazakhstan | *15,469* | *51* |  | *4358* | *34* | 1482 | 9.5790 |  | *977* | *15* | 147 | 0.9478 |  | 1679 | 10.8549 |
| Kyrgyzstan | *5,067* | *4* |  | *1788* | *34* | 608 | 11.9944 |  | *272* | *15* | 41 | 0.8063 |  | 653 | 12.8877 |
| Latvia | *2,329* | *2* |  | *376* | *34* | 128 | 5.4928 |  | *124* | *15* | 19 | 0.7964 |  | 148 | 6.3604 |
| Lithuania | *3,465* | *5* |  | *666* | *34* | 226 | 6.5338 |  | *146* | *15* | 22 | 0.6334 |  | 253 | 7.3146 |
| Luxembourg | *447* | *2* |  | *75* | *38* | 29 | 6.4129 |  | *28* | *44* | 12 | 2.7481 |  | 43 | 9.5964 |
| Malta | *393* | *1* |  | *16* | *38* | 6 | 1.5820 |  | *10* | *44* | 4 | 1.1279 |  | 12 | 2.9740 |
| Monaco | *34* | *0* |  | *4* | *38* | 2 | 4.6107 |  | *3* | *44* | 1 | 4.2707 |  | 3 | 9.1548 |
| **Netherlands** | *16,067* | *23* |  | *854* | *38* | 324 | 2.0196 |  | *511* | *44* | 225 | 1.4007 |  | 573 | 3.5655 |
| **Norway** | *4,514* | *7* |  | *240* | *38* | 91 | 2.0165 |  | *120* | *44* | 53 | 1.1704 |  | 151 | 3.3514 |
| **Poland** | *38,622* | *78* |  | *5601* | *34* | 1904 | 4.9309 |  | *2234* | *15* | 335 | 0.8677 |  | 2318 | 6.0005 |
| **Portugal** | *10,049* | *106* |  | *1896* | *38* | 721 | 7.1709 |  | *660* | *44* | 290 | 2.8880 |  | 1117 | 11.1158 |
| Moldova | *4,270* | *8* |  | *3809* | *34* | 1295 | 30.3234 |  | *294* | *15* | 44 | 1.0312 |  | 1347 | 31.5317 |
| **Romania** | *22,387* | *8* |  | *10996* | *34* | 3739 | 16.6993 |  | *2234* | *15* | 335 | 1.4970 |  | 4082 | 18.2332 |
| **Russian Federation** | *144,082* | *267* |  | *37426* | *34* | 12725 | 8.8317 |  | *8764* | *15* | 1315 | 0.9124 |  | 14306 | 9.9293 |
| San Marino | *27* |  |  | *3* | *38* | 1 | 4.2516 |  | *2* | *44* | 1 | 3.0607 |  | 2 | 7.3123 |
| Serbia and Montenegro | *10,535* | *13* |  | *1818* |  |  |  |  | *1060* |  |  |  |  | 13 | 0.1213 |
| Slovakia | *5,398* |  |  | *1313* | *34* | 447 | 8.2717 |  | *363* | *15* | 54 | 1.0094 |  | 501 | 9.2811 |
| Slovenia | *1,986* | *2* |  | *786* | *38* | 299 | 15.0367 |  | *135* | *44* | 60 | 3.0012 |  | 360 | 18.1357 |
| **Spain** | *40,977* | *687* |  | *5175* | *38* | 1966 | 4.7990 |  | *4826* | *44* | 2124 | 5.1822 |  | 4777 | 11.6587 |
| **Sweden** | *8,867* | *687* |  | *508* | *38* | 193 | 2.1750 |  | *532* | *44* | 234 | 2.6395 |  | 1114 | 12.5664 |
| **Switzerland** | *7,171* | *18* |  | *751* | *38* | 285 | 3.9779 |  | *525* | *44* | 231 | 3.2234 |  | 535 | 7.4549 |
| Tajikistan | *6,195* | *128* |  | *1303* | *34* | 443 | 7.1530 |  | *81* | *15* | 12 | 0.1950 |  | 583 | 9.4168 |
| Macedonia | *2,046* | *3* |  | *151* | *34* | 51 | 2.5111 |  | *210* | *15* | 31 | 1.5389 |  | 86 | 4.1890 |
| **Turkey** | *70,318* | *207* |  | *2602* | *34* | 885 | 1.2581 |  | *1162* | *15* | 174 | 0.2478 |  | 1266 | 1.7999 |
| Turkmenistan | *4,794* | *42* |  | *1421* | *34* | 483 | 10.0750 |  | *213* | *15* | 32 | 0.6650 |  | 557 | 11.6241 |
| Ukraine | *48,902* | *32* |  | *12459* | *34* | 4236 | 8.6622 |  | *2697* | *15* | 405 | 0.8273 |  | 4672 | 9.5541 |
| **United Kingdom** | *59,068* | *157* |  | *6465* | *38* | 2457 | 4.1590 |  | *2653* | *44* | 1167 | 1.9764 |  | 3781 | 6.4006 |
| Uzbekistan | *25,705* | *185* |  | *6695* | *34* | 2276 | 8.8548 |  | *692* | *15* | 104 | 0.4036 |  | 2565 | 9.9800 |
|  |  |  |  |  |  |  |  |  |  |  |  |  |  |  |  |
| **ALL** | **877,887** | **5205** |  | **170600** | **35** | **59971** | **6.8313** |  | **65811** | **32** | **21254** | **2.4210** |  | **86430** | **9.8453** |

Number of deaths are derived from WHO GBD data. Deaths due to hepatitis represent original WHO data, excluding liver cancer and cirrhosis deaths resulting from chronic HCV infection. Liver cancer and cirrhosis deaths attributable to HCV represent WHO GBD data weighted by population attributable fractions (AF). Population size data used to calculate death rates were derived from the WHO GBD project as well. Figures presented in italics represent external input data, figures in standard font indicate calculated results. Country names in bold indicate the 22 focus countries of our report.
Data sources:
a) WHO GBD data 2002 available at: http://www3.who.int/whosis/burden/estimates/2002Rev/2002RevCountries/DthDALY2002.zip
b) Population attributable fractions: Perz JF, Armstrong GL, Farrington LA, Hutin YJ and Bell BP. The contributions of hepatitis B virus and hepatitis C virus infections to cirrhosis and primary liver cancer worldwide. J Hepatol 2006;45(4):529-38

Table 4: DALYs and DALY rates related to HCV in countries of the WHO European region in 2002

|  |  | **HEPATITIS** |  | **CIRRHOSIS** | | | |  | **LIVER CANCER** | | | |  | **ALL** | |
| --- | --- | --- | --- | --- | --- | --- | --- | --- | --- | --- | --- | --- | --- | --- | --- |
| **Countries of the** | **Population** | **DALYS** |  | **Total** |  | **DALYS** | |  | **Total** |  | **DALYS** | |  | **DALYS** | |
| **WHO European Region** |  | **attr. to HCV** |  | **DALYS** | **AF** | **attr. to HCV** | |  | **DALYS** | **AF** | **attr. to HCV** | |  | **attr. to HCV** | |
|  | **('000)** | **n** |  | **n** | **%** | **n** | **per 100,000** |  | **n** | **%** | **n** | **per 100,000** |  | **n** | **per 100,000** |
| Albania | *3,141* | *81* |  |  | *34* |  |  |  | *4960* | *15* | 744 | 23.6883 |  | 825 | 26.2708 |
| Andorra | *69* | *7* |  | *134* | *38* | 51 | 73.8103 |  | *50* | *44* | 22 | 31.6535 |  | 80 | 115.8265 |
| Armenia | *3,072* | *35* |  | *6694* | *34* | 2276 | 74.0917 |  | *2656* | *15* | 398 | 12.9672 |  | 2710 | 88.2120 |
| **Austria** | *8,111* | *398* |  | *24341* | *38* | 9249 | 114.0353 |  | *5435* | *44* | 2391 | 29.4813 |  | 12039 | 148.4245 |
| Azerbaijan | *8,297* | *527* |  | *23463* | *34* | 7977 | 96.1512 |  | *5551* | *15* | 833 | 10.0361 |  | 9337 | 112.5361 |
| Belarus | *9,940* | *89* |  | *26913* | *34* | 9151 | 92.0596 |  | *3965* | *15* | 595 | 5.9839 |  | 9835 | 98.9423 |
| **Belgium** | *10,296* | *656* |  | *25332* | *38* | 9626 | 93.4966 |  | *4638* | *44* | 2041 | 19.8225 |  | 12323 | 119.6868 |
| Bosnia and Herzegovina | *4,126* | *97* |  | *8016* | *34* | 2725 | 66.0514 |  | *5583* | *15* | 837 | 20.2958 |  | 3659 | 88.6864 |
| Bulgaria | *7,965* | *263* |  | *20362* | *34* | 6923 | 86.9196 |  | *7398* | *15* | 1110 | 13.9329 |  | 8296 | 104.1517 |
| Croatia | *4,439* | *100* |  | *19749* | *38* | 7505 | 169.0782 |  | *3284* | *44* | 1445 | 32.5563 |  | 9049 | 203.8786 |
| Cyprus | *796* |  |  | *371* | *38* | 141 | 17.7280 |  | *423* | *44* | 186 | 23.3892 |  | 327 | 41.1173 |
| **Czech Republic** | *10,246* | *64* |  | *28833* | *38* | 10956 | 106.9362 |  | *7631* | *44* | 3358 | 32.7708 |  | 14378 | 140.3361 |
| **Denmark** | *5,351* | *85* |  | *14892* | *38* | 5659 | 105.7492 |  | *1912* | *44* | 841 | 15.7216 |  | 6586 | 123.0644 |
| Estonia | *1,338* | *20* |  | *5724* | *34* | 1946 | 145.4489 |  | *601* | *15* | 90 | 6.7375 |  | 2056 | 153.6581 |
| **Finland** | *5,197* | *160* |  | *12061* | *38* | 4583 | 88.1801 |  | *2578* | *44* | 1134 | 21.8241 |  | 5878 | 113.0885 |
| **France** | *59,850* | *1816* |  | *140576* | *38* | 53419 | 89.2549 |  | *53237* | *44* | 23424 | 39.1387 |  | 78659 | 131.4282 |
| Georgia | *5,177* | *7* |  | *21203* | *34* | 7209 | 139.2458 |  | *4055* | *15* | 608 | 11.7497 |  | 7824 | 151.1245 |
| **Germany** | *82,414* | *7971* |  | *264492* | *38* | 100507 | 121.9532 |  | *45230* | *44* | 19901 | 24.1475 |  | 128379 | 155.7728 |
| **Greece** | *10,970* | *445* |  | *7413* | *38* | 2817 | 25.6814 |  | *13269* | *44* | 5838 | 53.2245 |  | 9100 | 82.9596 |
| **Hungary** | *9,923* | *187* |  | *93358* | *34* | 31742 | 319.8883 |  | *7594* | *15* | 1139 | 11.4799 |  | 33068 | 333.2497 |
| Iceland | *287* | *4* |  | *82* | *38* | 31 | 10.8223 |  | *83* | *44* | 36 | 12.6654 |  | 71 | 24.7890 |
| **Ireland** | *3,911* | *124* |  | *2448* | *38* | 930 | 23.7904 |  | *1223* | *44* | 538 | 13.7567 |  | 1592 | 40.7147 |
| Israel | *6,304* | *735* |  | *3142* | *38* | 1194 | 18.9415 |  | *1486* | *44* | 654 | 10.3701 |  | 2583 | 40.9750 |
| **Italy** | *57,482* | *11195* |  | *110240* | *38* | 41891 | 72.8766 |  | *70278* | *44* | 30922 | 53.7947 |  | 84009 | 146.1472 |
| Kazakhstan | *15,469* | *1203* |  | *75553* | *34* | 25688 | 166.0636 |  | *9489* | *15* | 1423 | 9.2012 |  | 28314 | 183.0427 |
| Kyrgyzstan | *5,067* | *97* |  | *31659* | *34* | 10764 | 212.4291 |  | *2956* | *15* | 443 | 8.7506 |  | 11305 | 223.0992 |
| Latvia | *2,329* | *40* |  | *6419* | *34* | 2183 | 93.7094 |  | *1171* | *15* | 176 | 7.5413 |  | 2398 | 102.9673 |
| Lithuania | *3,465* | *84* |  | *12646* | *34* | 4300 | 124.0892 |  | *1175* | *15* | 176 | 5.0874 |  | 4560 | 131.6078 |
| Luxembourg | *447* | *12* |  | *1122* | *38* | 426 | 95.3598 |  | *211* | *44* | 93 | 20.7319 |  | 531 | 118.6912 |
| Malta | *393* | *11* |  | *242* | *38* | 92 | 23.4585 |  | *98* | *44* | 43 | 11.0163 |  | 146 | 37.2949 |
| Monaco | *34* | *1* |  | *62* | *38* | 23 | 68.5757 |  | *24* | *44* | 10 | 30.4847 |  | 35 | 102.4308 |
| **Netherlands** | *16,067* | *361* |  | *12723* | *38* | 4835 | 30.0908 |  | *4167* | *44* | 1833 | 11.4108 |  | 7029 | 43.7469 |
| **Norway** | *4,514* | *90* |  | *3671* | *38* | 1395 | 30.9093 |  | *754* | *44* | 332 | 7.3497 |  | 1817 | 40.2499 |
| **Poland** | *38,622* | *1097* |  | *93738* | *34* | 31871 | 82.5203 |  | *17908* | *15* | 2686 | 6.9552 |  | 35654 | 92.3152 |
| **Portugal** | *10,049* | *1656* |  | *29519* | *38* | 11217 | 111.6304 |  | *5502* | *44* | 2421 | 24.0930 |  | 15294 | 152.2001 |
| Moldova | *4,270* | *243* |  | *53605* | *34* | 18226 | 426.7966 |  | *3025* | *15* | 454 | 10.6249 |  | 18923 | 443.1102 |
| **Romania** | *22,387* | *162* |  | *159426* | *34* | 54205 | 242.1225 |  | *20090* | *15* | 3014 | 13.4608 |  | 57381 | 256.3088 |
| **Russian Federation** | *144,082* | *6158* |  | *677009* | *34* | 230183 | 159.7589 |  | *84009* | *15* | 12601 | 8.7460 |  | 248942 | 172.7788 |
| San Marino | *27* | *0* |  | *24* | *38* | 9 | 33.5571 |  | *14* | *44* | 6 | 23.0311 |  | 16 | 57.1033 |
| Serbia and Montenegro | *10,535* | *220* |  | *24606* |  |  |  |  | *9174* |  |  |  |  | 220 | 2.0876 |
| Slovakia | *5,398* |  |  | *22621* | *34* | 7691 | 142.4789 |  | *3207* | *15* | 481 | 8.9125 |  | 8172 | 151.3914 |
| Slovenia | *1,986* | *27* |  | *12094* | *38* | 4596 | 231.4278 |  | *1118* | *44* | 492 | 24.7736 |  | 5115 | 257.5791 |
| **Spain** | *40,977* | *6592* |  | *64195* | *38* | 24394 | 59.5307 |  | *32520* | *44* | 14309 | 34.9186 |  | 45295 | 110.5363 |
| **Sweden** | *8,867* | *417* |  | *6237* | *38* | 2370 | 26.7296 |  | *3136* | *44* | 1380 | 15.5628 |  | 4167 | 46.9904 |
| **Switzerland** | *7,171* | *185* |  | *10302* | *38* | 3915 | 54.5874 |  | *3767* | *44* | 1657 | 23.1094 |  | 5757 | 80.2770 |
| Tajikistan | *6,195* | *3707* |  | *23172* | *34* | 7878 | 127.1734 |  | *899* | *15* | 135 | 2.1771 |  | 11720 | 189.1861 |
| Macedonia | *2,046* | *37* |  | *2365* | *34* | 804 | 39.3128 |  | *1908* | *15* | 286 | 13.9942 |  | 1128 | 55.1223 |
| **Turkey** | *70,318* | *5913* |  | *50473* | *34* | 17161 | 24.4046 |  | *15060* | *15* | 2259 | 3.2126 |  | 25333 | 36.0261 |
| Turkmenistan | *4,794* | *1186* |  | *28496* | *34* | 9689 | 202.0972 |  | *2853* | *15* | 428 | 8.9275 |  | 11302 | 235.7542 |
| Ukraine | *48,902* | *919* |  | *224442* | *34* | 76310 | 156.0483 |  | *26395* | *15* | 3959 | 8.0963 |  | 81188 | 166.0233 |
| **United Kingdom** | *59,068* | *2414* |  | *114459* | *38* | 43494 | 73.6345 |  | *19702* | *44* | 8669 | 14.6764 |  | 54577 | 92.3978 |
| Uzbekistan | *25,705* | *5600* |  | *132821* | *34* | 45159 | 175.6793 |  | *9089* | *15* | 1363 | 5.3038 |  | 52122 | 202.7670 |
|  |  |  |  |  |  |  |  |  |  |  |  |  |  |  |  |
| **ALL** | **877,887** | **63496** |  | **2733544** | **35** | **957389** | **109.0561** |  | **532544** | **30** | **160219** | **18.2505** |  | **1181105** | **134.5395** |

Number of DALYs are derived from WHO GBD data. DALYs due to hepatitis represent original WHO data, excluding liver cancer and cirrhosis DALYs resulting from chronic HCV infection. Liver cancer and cirrhosis DALYS attributable to HCV represent WHO GBD data weighted by population attributable fractions (AF). Population size data used to calculate death rates were derived from the WHO GBD project as well. Figures presented in italics represent external input data, figures in standard font indicate calculated results. Country names in bold indicate the 22 focus countries of our report.
Data sources:
a) WHO GBD data 2002 available at: http://www3.who.int/whosis/burden/estimates/2002Rev/2002RevCountries/DthDALY2002.zip
b) Population attributable fractions: Perz JF, Armstrong GL, Farrington LA, Hutin YJ and Bell BP. The contributions of hepatitis B virus and hepatitis C virus infections to cirrhosis and primary liver cancer worldwide. J Hepatol 2006;45(4):529-38

Table 5: Transplantation – Number and proportion of liver transplants (LT) due to HCV related cirrhosis and liver cancer (LC) in countries of the WHO European region in 2004

|  |  |  | **CIRRHOSIS** | | | |  | **LIVER CANCER** | | | |  | **ALL** | |
| --- | --- | --- | --- | --- | --- | --- | --- | --- | --- | --- | --- | --- | --- | --- |
| **Countries of the** | **Total** |  | **LTs attr. to** | **Cirrhosis** | **LTs attr. to** | |  | **LTs attr. to** | **LC** | **LTs attr. to** | |  | **LTs attr. to** | |
| **WHO European Region** | **LTs** |  | **cirrhosis** | **attr. to HCV** | **HCV-Cirrhosis** | |  | **LC** | **attr. to HCV** | **HCV-LC** | |  | **HCV** | |
|  | **n** |  | **%** | **(AF) %** | **n** | **%** |  | **%** | **(AF) %** | **n** | **%** |  | **n** | **%** |
| Albania |  |  | *50* | *34* |  |  |  | *10* | *15* |  |  |  |  |  |
| Andorra |  |  | *50* | *38* |  |  |  | *10* | *44* |  |  |  |  |  |
| Armenia |  |  | *50* | *34* |  |  |  | *10* | *15* |  |  |  |  |  |
| Austria | *132* |  | *50* | *38* | 25 | 19 |  | *10* | *44* | 6 | 4 |  | 31 | 23 |
| Azerbaijan |  |  | *50* | *34* |  |  |  | *10* | *15* |  |  |  |  |  |
| Belarus |  |  | *50* | *34* |  |  |  | *10* | *15* |  |  |  |  |  |
| Belgium | *229* |  | *50* | *38* | 44 | 19 |  | *10* | *44* | 10 | 4 |  | 54 | 23 |
| Bosnia and Herzegovina |  |  | *50* | *34* |  |  |  | *10* | *15* |  |  |  |  |  |
| Bulgaria | *1* |  | *50* | *34* | 0 | 17 |  | *10* | *15* | 0 | 2 |  | 0 | 19 |
| Croatia | *43* |  | *50* | *38* | 8 | 19 |  | *10* | *44* | 2 | 4 |  | 10 | 23 |
| Cyprus |  |  | *50* | *38* |  |  |  | *10* | *44* |  |  |  |  |  |
| Czech Republic | *83* |  | *50* | *38* | 16 | 19 |  | *10* | *44* | 4 | 4 |  | 19 | 23 |
| **Denmark** | *42* |  | *50* | *38* | 8 | 19 |  | *10* | *44* | 2 | 4 |  | 10 | 23 |
| Estonia |  |  | *50* | *34* |  |  |  | *10* | *15* |  |  |  |  |  |
| Finland | *50* |  | *50* | *38* | 10 | 19 |  | *10* | *44* | 2 | 4 |  | 12 | 23 |
| **France** | *931* |  | *50* | *38* | 177 | 19 |  | *10* | *44* | 41 | 4 |  | 218 | 23 |
| Georgia |  |  | *50* | *34* |  |  |  | *10* | *15* |  |  |  |  |  |
| Germany | *881* |  | *50* | *38* | 167 | 19 |  | *10* | *44* | 39 | 4 |  | 206 | 23 |
| **Greece** | *29* |  | *50* | *38* | 6 | 19 |  | *10* | *44* | 1 | 4 |  | 7 | 23 |
| **Hungary** | *43* |  | *50* | *34* | 7 | 17 |  | *10* | *15* | 1 | 2 |  | 8 | 19 |
| Iceland |  |  | *50* | *38* |  |  |  | *10* | *44* |  |  |  |  |  |
| Ireland | *45* |  | *50* | *38* | 9 | 19 |  | *10* | *44* | 2 | 4 |  | 11 | 23 |
| Israel | *45* |  | *50* | *38* | 9 | 19 |  | *10* | *44* | 2 | 4 |  | 11 | 23 |
| Italy | *1035* |  | *50* | *38* | 197 | 19 |  | *10* | *44* | 46 | 4 |  | 242 | 23 |
| Kazakhstan |  |  | *50* | *34* |  |  |  | *10* | *15* |  |  |  |  |  |
| Kyrgyzstan |  |  | *50* | *34* |  |  |  | *10* | *15* |  |  |  |  |  |
| Latvia |  |  | *50* | *34* |  |  |  | *10* | *15* |  |  |  |  |  |
| Lithuania |  |  | *50* | *34* |  |  |  | *10* | *15* |  |  |  |  |  |
| Luxembourg |  |  | *50* | *38* |  |  |  | *10* | *44* |  |  |  |  |  |
| Malta |  |  | *50* | *38* |  |  |  | *10* | *44* |  |  |  |  |  |
| Monaco |  |  | *50* | *38* |  |  |  | *10* | *44* |  |  |  |  |  |
| Netherlands | *109* |  | *50* | *38* | 21 | 19 |  | *10* | *44* | 5 | 4 |  | 26 | 23 |
| **Norway** | *45* |  | *50* | *38* | 9 | 19 |  | *10* | *44* | 2 | 4 |  | 11 | 23 |
| **Poland** | *199* |  | *50* | *34* | 34 | 17 |  | *10* | *15* | 3 | 2 |  | 37 | 19 |
| **Portugal** | *205* |  | *50* | *38* | 39 | 19 |  | *10* | *44* | 9 | 4 |  | 48 | 23 |
| Moldova |  |  | *50* | *34* |  |  |  | *10* | *15* |  |  |  |  |  |
| Romania | *16* |  | *50* | *34* | 3 | 17 |  | *10* | *15* | 0 | 2 |  | 3 | 19 |
| **Russian Federation** |  |  | *50* | *34* |  |  |  | *10* | *15* |  |  |  |  |  |
| San Marino |  |  | *50* | *38* |  |  |  | *10* | *44* |  |  |  |  |  |
| Serbia and Montenegro |  |  | *50* |  |  |  |  | *10* |  |  |  |  |  |  |
| Slovakia |  |  | *50* | *34* |  |  |  | *10* | *15* |  |  |  |  |  |
| Slovenia | *15* |  | *50* | *38* | 3 | 19 |  | *10* | *44* | 1 | 4 |  | 4 | 23 |
| Spain | *1040* |  | *50* | *38* | 198 | 19 |  | *10* | *44* | 46 | 4 |  | 243 | 23 |
| **Sweden** | *133* |  | *50* | *38* | 25 | 19 |  | *10* | *44* | 6 | 4 |  | 31 | 23 |
| **Switzerland** | *84* |  | *50* | *38* | 16 | 19 |  | *10* | *44* | 4 | 4 |  | 20 | 23 |
| Tajikistan |  |  | *50* | *34* |  |  |  | *10* | *15* |  |  |  |  |  |
| Macedonia |  |  | *50* | *34* |  |  |  | *10* | *15* |  |  |  |  |  |
| Turkey | *245* |  | *50* | *34* | 42 | 17 |  | *10* | *15* | 4 | 2 |  | 45 | 19 |
| Turkmenistan |  |  | *50* | *34* |  |  |  | *10* | *15* |  |  |  |  |  |
| Ukraine |  |  | *50* | *34* |  |  |  | *10* | *15* |  |  |  |  |  |
| United Kingdom | *731* |  | *50* | *38* | 139 | 19 |  | *10* | *44* | 32 | 4 |  | 171 | 23 |
| Uzbekistan |  |  | *50* | *34* |  |  |  | *10* | *15* |  |  |  |  |  |
|  |  |  |  |  |  |  |  |  |  |  |  |  |  |  |
| ALL | **6411** |  |  |  | **1208** | **19** |  |  |  | **267** | **4** |  | **1475** | **23** |

Numbers of HCV attributable transplants are derived by weighting total liver transplant (LT) numbers by indication (cirrhosis, liver cancer (LC)) and HCV-attributable fraction (AF) for cirrhosis and liver cancer. Figures presented in italics represent external input data, figures in standard font indicate calculated results. Country names in bold indicate the 22 focus countries of our report.
Data sources:
a) Number of transplants: Transplant Committee of the Council of Europe. International Figures on organ donation and transplantation - 2004. Newsletter Transplant 2005;10(1):1-44
b) Distribution of transplant indications: Adam R, et al. Evolution of liver transplantation in Europe: Report of the European Liver Transplant Registry. Liver Transpl 2003;9(12):1231-43
c) HCV-attributable fractions for cirrhosis and liver cancer: Perz JF, Armstrong GL, Farrington LA, Hutin YJ and Bell BP. The contributions of hepatitis B virus and hepatitis C virus infections to cirrhosis and primary liver cancer worldwide. J Hepatol 2006;45(4):529-38
